# Decoding Molecular Network Dynamics in Cells: Advances in Multiplexed Live Imaging of Fluorescent Biosensors

**DOI:** 10.3390/bios15090614

**Published:** 2025-09-17

**Authors:** Qiaowen Chen, Yichu Xu, Jhen-Wei Wu, Jr-Ming Yang, Chuan-Hsiang Huang

**Affiliations:** 1 Department of Applied Mathematics and Statistics, Johns Hopkins University, Baltimore, MD 21218, USA; qchen82@jhu.edu; 2 Department of Biology, Johns Hopkins University, Baltimore, MD 21218, USA; 3 Department of Pathology, Johns Hopkins University, Baltimore, MD 21287, USA; yxu209@jh.edu (Y.X.); jwu223@jh.edu (J.-W.W.); 4 Department of Cell Biology, Johns Hopkins University, Baltimore, MD 21287, USA; 5 Center for Cell Dynamics, Johns Hopkins University, Baltimore, MD 21287, USA

**Keywords:** multiplexing, fluorescent biosensors, FRET, signaling network, live cell imaging, signaling dynamics

## Abstract

Genetically encoded fluorescent protein (FP)-based biosensors have revolutionized cell biology research by enabling real-time monitoring of molecular activities in live cells with exceptional spatial and temporal resolution. Multiplexed biosensing advances this capability by allowing the simultaneous tracking of multiple signaling pathways to uncover network interactions and dynamic coordination. However, challenges in spectral overlap limit broader implementation. Innovative strategies have been devised to address these challenges, including spectral separation through FP palette expansion and novel biosensor designs, temporal differentiation using photochromic or reversibly switching FPs, and spatial segregation of biosensors to specific subcellular regions or through cell barcoding techniques. Combining multiplexed biosensors with artificial intelligence-driven analysis holds great potential for uncovering cellular decision-making processes. Continued innovation in this field will deepen our understanding of molecular networks in cells, with implications for both fundamental biology and therapeutic development.

## 1. Introduction

Studying the molecular activities in cells is critical for advancing our understanding of cellular functions, disease mechanisms, and driving the development of therapeutics and biotechnology innovations. Probing changes in molecular activities within cells often require fixation or lysis, followed by the use of techniques such as biochemical quantification, immunoblotting, mass spectrometry, and nucleotide sequencing. While these methods are powerful and informative, they typically capture static snapshots of highly dynamic cellular systems, losing critical insights into the temporal and spatial dynamics of molecular activities, which play important roles in determining cellular outcomes [1]. To address this limitation, researchers have developed methods for tracking molecular processes in live cells based on techniques such as fluorescence and bioluminescence imaging, Raman microscopy, and electrophysiology techniques. These tools enable real-time monitoring of molecular events while preserving the native environment of the cells.

Among live-cell technologies, genetically encoded fluorescent biosensors have emerged as powerful tools due to their exceptional versatility, specificity, and ability to provide dynamic insights into cellular processes [2,3,4,5,6,7]. As their name suggests, these biosensors are encoded by exogenous DNA sequences that produce proteins capable of emitting fluorescent signals. The majority of biosensors rely on fluorescent proteins (FPs) as the source of their fluorescence. They also contain a sensing module that responds to specific biochemical events, such as changes in post-translational modifications, ion concentration, pH, enzyme activity, or protein–protein interactions, leading to altered localization, intensity, or spectrum of the fluorescence.

Fluorescent biosensors provide critical insights into the spatiotemporal activity of biomolecules, enabling observations that are challenging to achieve with other methods. These dynamic behaviors have been observed across multiple scales, from subcellular regions to whole cell populations, and are essential to a wide range of biological functions. For example, ERK (extracellular signal-regulated kinase) activity is pulsatile at the single-cell level triggered by localized Ras (rat sarcoma virus protein) activation on protrusions, with pulse frequency regulating cell proliferation [8,9,10], while ERK waves coordinate collective cell migration during development and tissue repair [11,12,13,14,15]. Oscillatory signaling through pathways such as Notch, p53, and NF-κB (nuclear factor kappa-light-chain-enhancer of activated B cells) governs processes like embryonic segmentation and cellular stress responses [16,17,18]. Additionally, certain biomolecular activities propagate as waves either within individual cells or across cell populations. For instance, Ras-PI3K (phosphoinositide 3-kinase) waves drive actin remodeling and cell motility in amoeboid and epithelial cells [19,20,21,22,23], and MinCDE waves in bacteria facilitate the positioning of division machinery [24,25]. Disruptions in these signaling behaviors are associated with diseases, including cancer [26,27,28,29].

## 2. Types of Genetically Encoded Fluorescent Biosensors

**Figure 1:**
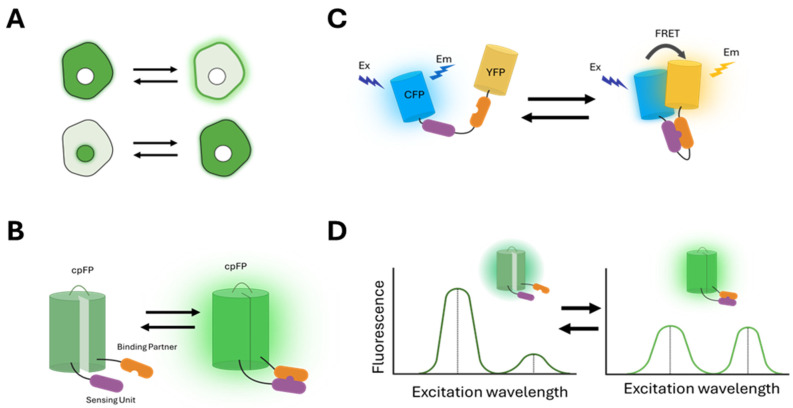
**Readouts of genetically encoded fluorescent biosensors**. Biosensor readouts are often based on changes in (**A**) intracellular localization, (**B**) fluorescence intensity, (**C**) FRET efficiency, or (**D**) spectral profiles (as illustrated by an excitation ratiometric biosensor).

While fluorescent biosensors are designed with diverse molecular detection mechanisms, the readouts commonly fall into the following categories:

(1) Changes in the localization of the fluorescence signal (Figure 1A): These sensors undergo translocation to different cellular compartments through binding to specific molecules or conformational changes involving localization signals. For example, the pleckstrin homology (PH) domain of the AKT (Ak strain transforming) kinase, PH-AKT, binds to phosphatidylinositol (3,4,5)-triphosphate (PIP3) that accumulates at the plasma membrane when PI3K is activated. Therefore, FP-tagged PH-AKT is used as a sensor for PI3K activity [30]. Another example is kinase translocation reporters (KTRs), which combine a nuclear localization signal (NLS), a nuclear export signal (NES), and kinase-specific phosphorylation sites within a single polypeptide. Phosphorylation by the target kinase alters the affinity of these sensors for importins and exportins, leading to a change in subcellular localization that can be detected as fluorescence redistribution [31,32,33,34].

(2) Changes in fluorescence intensity (Figure 1B): These sensors change the fluorescence intensity in response to specific molecular events. In particular, many of them include a circularly permuted GFP (cpGFP) that alters its fluorescence upon conformational changes induced by the binding of target molecules [35]. Examples include the GCaMP6 (genetically encoded calcium indicator version 6) family sensors for calcium [36] and GRAB (G protein-coupled receptor-activation-based) family sensors for neuropeptides [37,38].

(3) Changes in the FRET (Förster resonance energy transfer) between two FPs (Figure 1C): FRET is the non-radiative transfer of energy from a donor fluorophore to a nearby acceptor fluorophore [39,40]. A widely used approach in biosensor design leverages conformational changes that affect the distance or relative orientation between donor and acceptor FPs, resulting in variations in FRET efficiency [4,41,42]. Examples include many kinase activity reporters, which consist of a substrate specific to the target kinase and a binding domain that recognizes the phosphorylated form of the substrate, with donor and acceptor FPs positioned on either side [5,43,44,45,46,47,48]. Phosphorylation of the substrate by the target kinase induces an intramolecular binding event that modifies FRET, detectable through various techniques such as changes in the fluorescence intensity ratio between the donor and acceptor, donor fluorescence after acceptor photobleaching, donor fluorescence lifetime, or fluorescence depolarization [49].

(4) Changes in the spectral profiles of fluorescence (Figure 1D): These sensors exhibit different excitation or emission profiles in response to specific molecular events. For example, excitation ratiometric biosensors use the ratio of fluorescence intensity excited at two different wavelengths as the readout for specific activities [50,51,52,53].

## 3. Multiplexing Fluorescent Biosensors

Biological processes depend on the coordinated and synergistic activities of networks of signaling proteins, second messengers, metabolites, ion fluxes, and other molecular players. While individual biosensors have provided unprecedented insights into the spatiotemporal dynamics of these molecules, they fall short of capturing the complex interactions between them. In recent years, there has been growing interest in the simultaneous imaging of multiple biosensors, which offers a powerful approach to uncover the interactions between these different components in real time and within their native cellular environments [54,55,56].

A main challenge in multiplexed imaging of biosensors is the spectral overlap between FPs. Due to the limited availability of spectral space, many biosensors employ FPs with overlapping excitation or emission spectra, which complicates the unambiguous resolution of individual signals. To address this issue, the ongoing expansion of the FP palette has introduced brighter, more photostable, and spectrally distinct FPs, offering greater flexibility in biosensor design for simultaneous imaging. Moreover, innovative imaging techniques coupled with breakthroughs in computational tools and machine learning algorithms have further increased the multiplexing capacity and facilitated the interpretation of complex datasets.

In general, multiplexing biosensors require the ability to resolve fluorescence signals based on distinct spectral, temporal, or spatial properties. In the following sections, we review recent advancements that leverage these principles.

## 4. Spectral Multiplexing

By carefully selecting FPs with minimal spectral overlap, multiple biosensors can be expressed in the same cells and imaged simultaneously. The degree of multiplexing achievable depends on the extent of spectral separation between the biosensors used. In general, biosensors containing a single fluorophore allow for higher multiplexing compared to those that use fluorophore pairs, such as FRET-based biosensors.

### 4.1. Single-Fluorophore Biosensors

**Figure 2:**
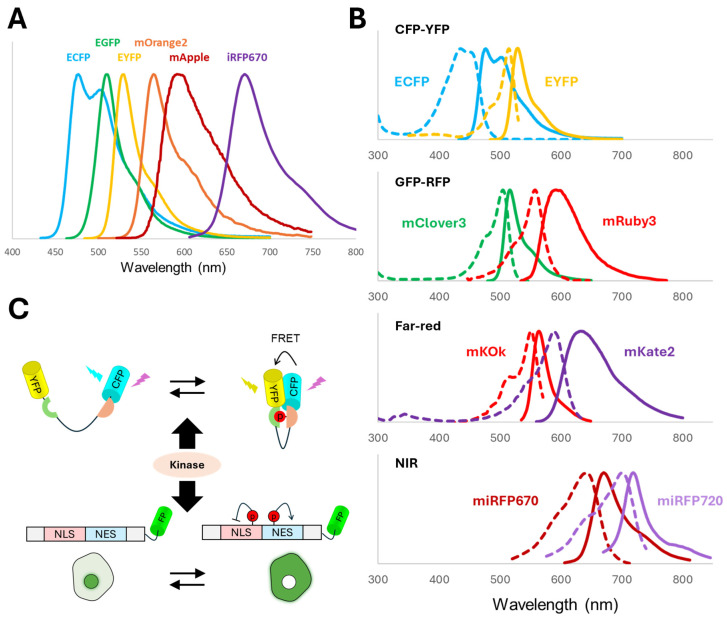
**Spectral multiplexing.** (**A**) Emission spectra of representative FPs. (**B**) Excitation (dotted lines) and emission (solid lines) spectra of different FRET pairs: cyan–yellow (ECFP-EYFP), green–red (mClover3-mRuby3), far-red (mKOκ-mKate2), and near-infrared (miRFP670-miRFP720). The spectral data were downloaded from FPbase [59]. (**C**) Conversion of a FRET-based kinase activity reporter (top) into kinase translocation reporter (bottom).

Single-FP biosensors with minimal spectral overlap can be readily distinguished by selecting appropriate emission ranges during image acquisition. In particular, yellow or green FPs are often combined with red FPs to achieve dual-biosensor imaging (Figure 2A). This can be applied to biosensors based on both conventional and circularly permuted FPs. For example, cpGFP and cpmApple (circularly permuted monomeric Apple) were used to create highly sensitive biosensors for simultaneous live monitoring of extracellular and intracellular levels of lactate, an important metabolic and signaling molecule in diverse physiological and pathological processes [57]. In another example, dual color imaging of a yellow cAMP (cyclic adenosine monophosphate) sensor and a red calcium sensor was used to reveal the distinct kinetic responses of cAMP and calcium to noradrenaline stimulation [58].

To achieve higher levels of multiplexing, FPs with significant spectral overlaps can be distinguished using spectral imaging followed by linear unmixing. This method assumes that the total measured fluorescence at each wavelength is a linear combination of signals from all fluorophores present. By referencing the known emission spectra of individual fluorophores, linear unmixing can determine the relative contribution of each fluorophore to the overall measured signal. This strategy has been shown to enable simultaneous imaging of up to five or six different fluorophores (Figure 2A) [60].

In addition to FP-based biosensors, chemigenetic biosensors offer versatile protein labeling by using self-labeling protein tags that specifically bind exogenous synthetic fluorophores [61]. Examples of such protein tags include HaloTag [62], SNAP-tag [63], CLIP-tag [64], PYP-tag [65], FlAsH [66], ReAsH [67], eDHFR/TMP [68], and FAST [69]. Compared to FPs, synthetic fluorophores used with these tags may have narrower emission spectra, which minimizes spectral overlap and facilitates multiplexed imaging. They also offer enhanced signal-to-noise ratios and greater photostability, supporting advanced imaging techniques such as super-resolution microscopy, single-molecule tracking, and long-term live-cell imaging.

For example, a series of highly sensitive chemigenetic biosensors for PKA (protein kinase A), PKC (protein kinase C), AKT, and ERK were developed by integrating a kinase-specific sensing unit and a phosphoamino acid-binding domain with a circularly permuted HaloTag (cpHaloTag) reporting unit that is labeled by a far-red dye [70]. By co-expressing biosensors for PKA (HaloAKAR2.2-JF635), cAMP (pinkFlamindo33), Ca2+ (B-GECO1), and PKC (ExRai-CKAR2) activities, signaling dynamics by distinct G protein classes were simultaneously monitored [70]. The study showed that both Gs- and Gq/11-coupled GPCRs (G protein-coupled receptors) can activate Ca^2+^, PKC, cAMP, and PKA pathways, generating dynamic, cell-specific signaling responses to ligands and drugs [70]. These findings challenge the traditional view that GPCRs signal through linear pathways by coupling exclusively to a single G protein class, instead revealing extensive crosstalk with implications for disease mechanisms and therapeutic strategies.

### 4.2. FRET-Based Biosensors

FRET between two FPs is a generalizable strategy used for the construction of many biosensors [41,49]. These dual-FP biosensors occupy a larger spectral space than single-FP biosensors, thus limiting the multiplicity. The most commonly used FRET pair is the CFP-YFP (e.g., ECFP-EYFP) pair (Figure 2B). More recently, GFP-RFP FRET pairs (e.g., mClover-mRuby) have been developed and suggested to have better spectral separation, greater dynamic range, and less phototoxicity than the CFP-YFP pair [49,71,72]. However, the spectral overlap between CFP-YFP and GFP-RFP pairs makes them unsuitable for simultaneous imaging (Figure 2B). To address this issue, far-red and near-infrared (NIR) FRET pairs have been successfully used to co-image the CFP-YFP pair [73,74]. While the boundaries are not strict, far-red FPs typically refer to GFP-like proteins derived from cnidarians with peak excitation between 600 and 650 nm, whereas NIR FPs are often bacterial phytochromes with peak excitation above 650 nm and require an exogenous chromophore such as biliverdin [75,76,77].

An example of the far-red FRET pair is the mKOκ-mKate2 pair (Figure 2B), which was used to create a series of sensors for ERK, JNK (c-Jun N-terminal kinase), and ROCK (Rho-associated coiled-coil kinase). These sensors can be co-imaged with CFP-YFP FRET sensors [73]. An example of the NIR FRET pair is miRFP670-miRFP720 (Figure 2B). A Rac1 (Ras-related C3 botulinum toxin substrate 1) biosensor based on miRFP670-miRFP720 FRET was simultaneously imaged with a CFP-YFP RhoA (Ras homolog family member A) biosensor to reveal the Rac1-RhoA antagonism in motile cells [78]. The NIR Rac1 biosensor was also imaged with a CFP-YFP-based Rac1-GDI (GDP dissociation inhibitor) biosensor, demonstrating that both active Rac1 and GDI-inactivated Rac1 can simultaneously localize to edge protrusions [78]. miRFP670-miRFP720-based biosensors for the signaling protein ERK have also been developed [79].

FRET pairs that share a common acceptor but differ in donor excitation spectra can be sequentially imaged, thus enabling more efficient use of limited spectral space to allow for higher multiplexity. For example, CFP-RFP (Cerulean–mCherry) and YFP-RFP (mVenus-mCherry) both undergo FRET efficiently. These pairs were used to develop the FRET-based biosensors CR-AKAR and YR-ICUE, enabling the simultaneous monitoring of PKA and cAMP signaling in live single cells [80]. Co-imaging these biosensors within the same cell revealed distinct response profiles to various GPCR agonists, allowing for precise dissection of pathway-specific signaling dynamics. In addition, simultaneous tracking of kinases Src, Akt, and ERK was achieved using biosensors based on three FRET pairs: red–far-red (smuRFP-stagRFP), yellow–red (Venus–stagRFP) and green–red (T-sapphire–stagRFP) [81].

During FRET, the excited state of the donor undergoes faster quenching, resulting in a shorter fluorescence lifetime. This property has been exploited in fluorescence-lifetime imaging microscopy (FLIM) to directly measure donor fluorescence lifetime. Requiring specialized imaging systems, FLIM eliminates the need for measuring acceptor emission and enables the use of weakly fluorescent acceptor proteins, thus freeing up emission spectra for other FPs and facilitating multiplexing [55]. Using this approach, an ERK FLIM-FRET reporter using donor mTFP (monomeric teal fluorescent protein) paired with a dim fluorescent acceptor ShadowG (mTFP-ShadowG) was co-imaged with an AKAR biosensor based on the LSSOrange-mKate2 FRET pair. Exciting both donors with a single laser allowed simultaneous monitoring of ERK and PKA activities in cells stimulated with EGF (epidermal growth factor) [82].

### 4.3. Converting FRET-Based Biosensors into Single-FP Biosensors

Despite the expanded repertoire of FRET pairs, it remains challenging to simultaneously image more than two or three FRET biosensors. To overcome this limitation, significant efforts have been made to develop single-FP formats as alternatives to sensors that were originally based on FRET [83]. For instance, the KTR family of biosensors exploits nucleocytoplasmic shuttling in response to the activation of specific protein kinases (Figure 2C) [31,32,33]. By tagging these KTRs with different FPs, researchers have enabled simultaneous tracking of key signaling proteins, such as mitogen-activated protein kinases (MAPKs), PKA, and AKT, revealing their distinct kinetic responses to specific inhibitors.

Another example is the excitation ratiometric (ExRai) biosensor family, which leverages the shifting excitation profiles of certain cpFPs possibly due to changes between the chromophore in different protonation states. This approach enables the use of signal intensity ratios from a single fluorophore measured at two distinct excitation wavelengths as the readout [53,84,85,86]. Using ExRai biosensors, researchers successfully achieved fourfold multiplexed imaging of signaling pathways involving PKA, cAMP, PKC, and Ca^2+^. Furthermore, by localizing these biosensors to specific subcellular compartments, it was possible to simultaneously monitor up to six separate signaling activities within individual cells [53].

FLuorescence Anisotropy REporters (FLAREs) represent yet another strategy by exploiting homo-FRET, i.e., the energy transfer between identical fluorophores. Homo-FRET leads to loss of polarization, or anisotropy, of emitted light, which is used as a readout. This enables each FRET biosensor to use only a single FP. By engineering FLARE biosensors with spectrally distinct fluorophores, researchers were able to simultaneously track PKA, ERK, and calcium signaling in HEK239T cells stimulated with forskolin, EGF, and thapsigargin [87]. Furthermore, homo-FRET can be combined with hetero-FRET in a binary FRET approach, where readouts are based on anisotropy changes (homo-FRET) and fluorescence lifetime measurements (hetero-FRET) [88].

## 5. Temporal Multiplexing

**Figure 3:**
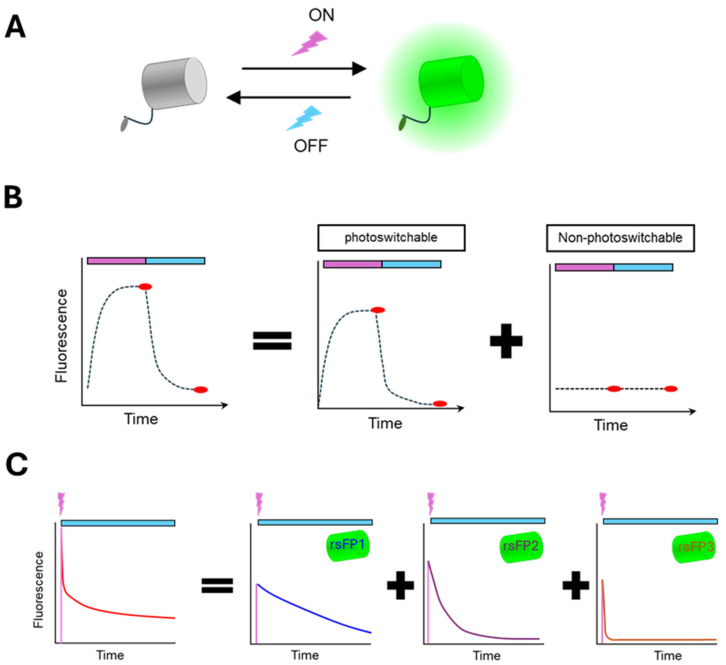
**Temporal multiplexing**. (**A**) Reversible photoswitching of the fluorescent protein achieved by using two excitation wavelengths to turn on (purple) or turn off (blue) the fluorescence. (**B**) Multiplexing photochromic and non-photochromic biosensors. Two illumination periods are used to turn on (purple) and turn off (blue) the photochromic biosensors. Red ovals indicate when images are acquired. (**C**) Temporally multiplexed imaging of reversibly photoswitchable biosensors with distinct off-switching kinetics. Biosensors are switched on by a short period of excitation (purple) followed by illumination with the off-switching light (blue). Images are continuously acquired once the off-switching light is on.

Time-dependent fluorescent properties of FPs can be leveraged to differentiate biosensors with similar emission spectra. This approach often utilizes photochromic FPs, which exhibit reversible changes in fluorescence intensity or spectrum when exposed to specific wavelengths of light [89] (Figure 3A). For example, a FRET biosensor containing a reversibly photoswitchable FP (rsFP) donor can be co-imaged with a non-photochromic FRET sensor. By analyzing the reaction to off- and on-switching light, the contribution from each biosensor can be determined (Figure 3B). This strategy was used to visualize two FRET biosensors, for ERK and PKA in the same cells, and combined with the red-shifted calcium sensor RCaMP for three-fold multiplexing [90]. Their results revealed heterogeneity in cellular responses and demonstrated the potential to classify cells according to differences in their signaling activities.

To provide greater scalability, the temporally multiplexed imaging (TMI) technique employs rsFPs with distinct off-switching rates [91]. The signals from different rsFP biosensors with similar emission spectra can be unmixed based on characteristic decay kinetic patterns induced by off-switching light (Figure 3C). Using this approach, six green FPs were expressed for simultaneous imaging. By combining temporal and spectral multiplexing principles, seven biosensors (four green, one near-infrared, one red, one blue) were successfully co-imaged. This strategy was used to identify cyclin-dependent kinase 2 (CDK2) and CDK4/6 dynamics throughout different cell cycle phases, and to distinguish two distinct ERK response types following bFGF (basic fibroblast growth factor) stimulation, potentially through interactions with JNK, PKA, and p38 signaling pathways [91].

## 6. Spatial Multiplexing

### 6.1. Targeting Biosensors to Different Subcellular Compartments

**Figure 4:**
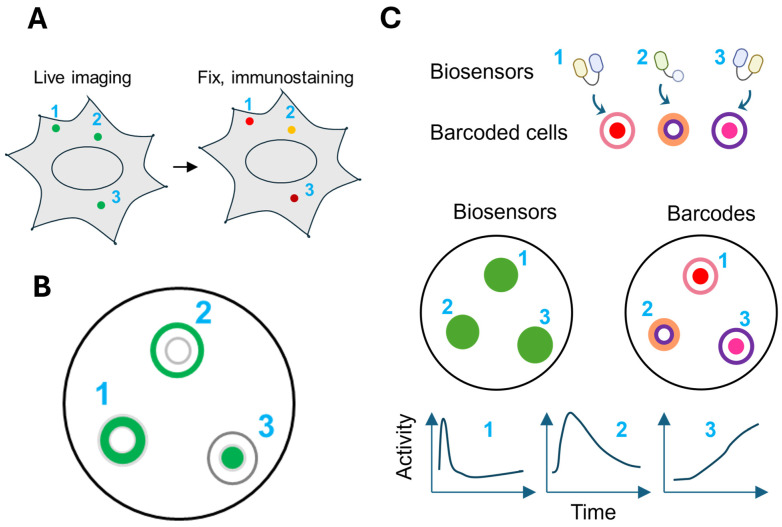
**Spatial multiplexing**. (**A**) The signaling reporter islands method. (**B**) Targeting fluorescent biosensors to different subcellular locations. (**C**) Multiplexed biosensor imaging using barcoded cells expressing biosensors. Biosensor (numbered 1–3) signals are shown in green.

Biosensors with overlapping spectra can also be distinguished by employing distinct spatial patterns to separate their signals. For example, biosensors targeted to different subcellular locations in the same cell can be identified by their spatial distribution within the cell. However, such localization is often imperfect, leading to signal overlap and interference between sensors. To overcome this problem, in the signaling reporter islands (SiRIs) method, sensors are clustered into bright puncta by fusing to pairs of self-assembling peptides [92] (Figure 4A). The signals from different puncta can be resolved using confocal microscopy. Each biosensor is also fused to a unique epitope tag, allowing its identity to be determined post hoc via immunostaining after live imaging. Simultaneous observation of three SiRIs revealed that hippocampal neurons with faster calcium responses exhibit larger downstream PKA activation [92].

A key advantage of multiplexing biosensors within the same cell is that it can reveal dynamic relationships between various molecular activities that may be masked in ensemble measurements. However, the physical and functional capacity of a single cell poses a practical limitation on the number of biosensors that can be simultaneously expressed. As more biosensors are introduced, the risk of cellular stress and toxicity increases, potentially disrupting normal cellular processes. Another important consideration is the potential interference between biosensors, which might confound the interpretation of reported activities in sensitive systems.

Instead of localizing biosensors to different compartments within the same cell, an alternative strategy for spatial multiplexing is to express sensors in separate cells. This approach prevents signal and functional interference between biosensors, and reduces cellular toxicity caused by the expression burden associated with co-expressing multiple biosensors in a single cell. In one example, FRET biosensors for RTK (receptor tyrosine kinase)/Ras/PI3K/MAPK signaling targeted to three different subcellular compartments (plasma membrane, nucleus, and cytosol) were expressed in separate cells, which were then mixed for simultaneous imaging (Figure 4B). The identity of the sensor in each cell was determined by its localization pattern. Using this method, the dynamics of feedforward and feedback regulation within the signaling network and their roles in the resistance of cancer cells to MEK (mitogen-activated protein kinase kinase) and PI3K inhibitors were delineated [93]. Similarly, this strategy was also applied to image the H_2_O_2_ biosensor HyPer7 targeted to the nucleus, mitochondria, and cytosol, revealing that the mitochondria and plasma membrane exhibit faster and stronger oxidation, while the nucleus displays more buffered responses [94].

### 6.2. Multiplexing Biosensors Using Barcoded Cells

Spatial multiplexing by targeting biosensors to different subcellular compartments comes with two key limitations. First, the total number of biosensors is constrained by the number of compartments that can be reliably distinguished in cell images, typically no more than four or five. Second, depending on the diffusion rate and kinetics of the reported activity, anchoring a biosensor to a specific location generally limits the measurement of activity to that site.

A strategy to address these limitations is to decouple the biosensor from its spatial identity by introducing a separate barcoding system [95,96,97,98]. In the “biosensor barcoding” method, each biosensor is co-expressed with a unique visual barcode made of spectrally orthogonal FPs. Barcoded cells expressing different biosensors are mixed for simultaneous imaging, and the identity of the sensor in each cell is determined by its barcode (Figure 4C). A key advantage of this strategy is that it can support the simultaneous detection of as many biosensors as there are unique barcodes, thus greatly expanding the multiplexing capacity. Moreover, any existing biosensor can be readily incorporated in the scheme without the need for further engineering, provided it is spectrally separable from the barcoding FPs. Expressing different biosensors in separate cells also reduces the biosensor interference, cellular toxicity, or signal crosstalk that can arise when multiple sensors are co-expressed in a single cell. However, an important trade-off is that these strategies do not allow direct correlation of multiple signaling pathways within the same individual cell.

There are different ways to generate barcodes. In one strategy, barcodes comprise two FPs targeted to different subcellular locations. Using four FPs (one blue FP and three red FPs) targeted to four subcellular locations (the nucleus, cytoplasm, nuclear membrane, and cytosol), 72 distinct barcodes can be generated to support highly multiplexed imaging of CFP/GFP/YFP biosensors, including those based on CFP-YFP FRET [95]. Barcode identification is facilitated by machine learning models trained on cells expressing known barcodes [95,96]. Another strategy for barcode generation involves linking different FPs into a polypeptide chain. These single-chain tandem FP (sctFP) barcodes can be identified by the ratio between the fluorescence signals from different FPs. These barcodes have lower demand for image resolution compared to location-based barcodes and can support a broader range of biosensor spectrum due to their robustness [99].

The high degree of multiplexing enabled by the biosensor barcoding technology allows for comprehensive tracking of signaling network dynamics. For example, simultaneous monitoring of 24 biosensors for various kinase and G protein signaling pathways uncovered several unexpected responses, suggesting potential interactions between these pathways. Moreover, focusing on biosensors for different nodes of the RTK signaling network, their responses to varying doses of EGF stimulation exhibited distinct dynamic patterns and cooperativity among downstream pathways, indicative of diverse topological organizations. Systematic perturbation of individual nodes within the network further revealed complex feedback loops between these nodes [95].

In addition to studying signaling network dynamics, barcodes can be used to label different cell populations to investigate their signaling interactions. For example, monitoring RTK network biosensors in co-cultured cells with or without mutant KRAS (Kirsten rat sarcoma viral oncogene homologue) revealed cell non-autonomous effects of the KRAS mutation, mediated through metalloproteinases [95]. Finally, barcodes can be leveraged to introduce calibration standards into a subset of labeled cells, allowing correction for variations in biosensor signals caused by fluctuations in imaging conditions. This enables precise, calibrated, and multiplexed tracking of biosensors over time [100].

### 6.3. Other Approaches

By also employing spatial separation strategy, the MOSAIC (multiplexed optical sensors in arrayed islands of cells) technique used a microarray printer to deposit lentiviruses encoding different sensors onto patterned fibronectin islands on a cell-repellent substrate. Seeded cells only adhere to the fibronectin-coated region and become transduced by the lentivirus to express the specific sensor. Different biosensors are expressed in cells in the same well and experience the same intervention, thus avoiding well-to-well variability. The biosensor identity is encoded by the position of the cell cluster in the dish. This enabled up to 20 sensors sharing similar spectrum to be imaged simultaneously [101].

## 7. Conclusions and Future Directions

Biosensors have revolutionized the study of cellular mechanisms, enabling high-resolution imaging and the investigation of subcellular processes with remarkable precision. They not only shed light on intracellular phenomena in real time but also reveal the heterogeneity among individual cells that is often masked in bulk ensemble measurements. The ability to simultaneously monitor numerous molecular activities in live cells has substantially advanced our understanding of complex cellular behaviors in both normal and diseased states.

This growing capacity to track molecular activities at high spatiotemporal resolution in parallel generates vast amounts of data, presenting new challenges and opportunities. Unlocking the full potential of this data requires the development of more sophisticated computational tools for image processing, analysis, and interpretation. Importantly, this data offers an unprecedented opportunity to construct quantitative models of molecular networks [102], enabling deeper insights into cellular systems and improved predictions of cellular responses to external stimuli, such as drugs. Looking ahead, integrating artificial intelligence and machine learning offers immense potential for designing new biosensors [103,104] and enhancing the extraction of meaningful patterns from complex datasets [105], driving novel discoveries and therapeutic innovations.

While biosensors provide invaluable insights into cellular processes, their application is not without challenges. Overexpression of biosensors can disrupt normal cellular physiology, potentially compromising the accuracy of experimental findings [106,107,108]. Imaging biosensors may expose cells to intense or prolonged light irradiation, particularly when low fluorescence signals require higher illumination, leading to phototoxicity that can damage cellular components or alter biological functions [108]. FPs vary in their photostability and are sensitive to pH levels [109], which may further affect data quality. Additionally, some biosensors are limited by a narrow dynamic range, constraining their ability to accurately detect events outside their optimal sensitivity. Addressing these issues requires rigorous experimental design, including careful calibration, appropriate baseline controls, and complementary validation methods to confirm findings. Encouragingly, recent advances in biosensor technology have addressed many of these limitations by improving their brightness, photostability, and dynamic range, thus broadening their utility across a wider array of experimental settings.

With ongoing improvements in biosensor design, including increased sensitivity, multiplexed imaging capabilities, and the integration of advanced computational approaches, the potential of these tools continues to grow. They promise to bridge the gap between fundamental research and clinical applications, providing new avenues for diagnosing, monitoring, and treating diseases with exceptional precision. As these technologies evolve, their impact will undoubtedly extend across many domains of biology and medicine.

## Data Availability

No new data were created or analyzed in this study.

## Abbreviations

AKT	Ak strain transforming
bFGF	basic fibroblast growth factor
cAMP	cyclic adenosine monophosphate
CDK	cyclin-dependent kinase
CFP	cyan fluorescent protein
cpGFP	circularly permuted green fluorescent protein
EGF	epidermal growth factor
ERK	extracellular signal-regulated kinase
ExRai	excitation ratiometric
FLARE	fluorescence anisotropy reporter
FLIM	fluorescence lifetime imaging microscopy
FP	fluorescent protein
FRET	Förster resonance energy transfer
GCaMP	genetically encoded calcium indicator
GDI	GDP dissociation inhibitor
GPCR	G protein-coupled receptor
GRAB	G protein-coupled receptor-activation-based sensor
JNK	c-Jun N-terminal kinase
KRAS	Kirsten rat sarcoma viral oncogene homologue
KTR	kinase translocation reporter
MAPK	mitogen-activated protein kinase
MEK	mitogen-activated protein kinase kinase
NF-κB	nuclear factor kappa-light-chain-enhancer of activated B cells
NES	nuclear export signal
NLS	nuclear localization signal
PH	pleckstrin homology
PI3K	phosphoinositide 3-kinase
PKA	protein kinase A
PKC	protein kinase C
Rac1	Ras-related C3 botulinum toxin substrate 1
Ras	rat sarcoma virus protein
RFP	red fluorescent protein
RhoA	Ras homolog family member A
ROCK	Rho-associated coiled-coil kinase
rsFP	reversibly photoswitchable fluorescent protein
RTK	receptor tyrosine kinase
TFP	teal fluorescent protein
YFP	yellow fluorescent protein
